# HAND-FOOT SYNDROME DUE TO CAPECITABINE

**DOI:** 10.4103/0019-5154.55651

**Published:** 2009

**Authors:** Amar Surjushe, Resham Vasani, Sudhir Medhekar, Minal Thakre, D G Saple

**Affiliations:** *From the Department of Dermatology, Venereology, & Leprology, Grant Medical College and Sir JJ Groups of Hospitals, Mumbai - 400 008, India.*; 1 *From the Department of Pathology, Grant Medical College and Sir JJ Groups of Hospitals, Mumbai - 400 008, India. E-mail: dramarsurjushe@rediffmail.com*

Sir,

Hand-foot syndrome, also known as Palmar-plantar erythrodysesthesia is a side effect, which mostly occurs with chemotherapy or biologic therapy. In mild to moderate cases there may be painful erythema and edema, various degrees of dysesthesia, which may be followed by dry or moist desquamation of the palms and the soles. In more severe cases, there may be cracking, flaking, peeling of skin, blisters, ulcers and severe pain. These may interfere with the daily activities.[1]

It was first reported by Lokich and Moore in 1984 with 5-FU.[2] Drugs that have been associated include 5-flurouracil, capecitabine, cytarabine, doxorubicin, epirubicin, high-dose interleukin-2, fluorodeoxyuridine (FUDR), hydroxyurea, mercaptopurine, cyclophosphamide, and docetaxel.

We document a case of hand-foot syndrome caused by capecitabine. A 50-year- old female patient was operated for moderately differentiated adenocarcinoma of the common bile duct (cholangiocarcinoma). Post procedural ultrasound of the abdomen was suggestive of a SOL of altered echotexture measuring 2.3 cm in the left lobe of liver and a mixed echogenic mass of size 4 cm in the right ovary. Positron Emission Tomography scan and MRI – Fluorodeoxyglucose scan was suggestive of metastasis. Multiple nodal metastases were seen in the peripancreatic, gastrosplenic, cardiophrenic, celiac, aortocaval, supraclavicular, right axillary node and in the abdominal wall on both sides.

**Figure 1:**
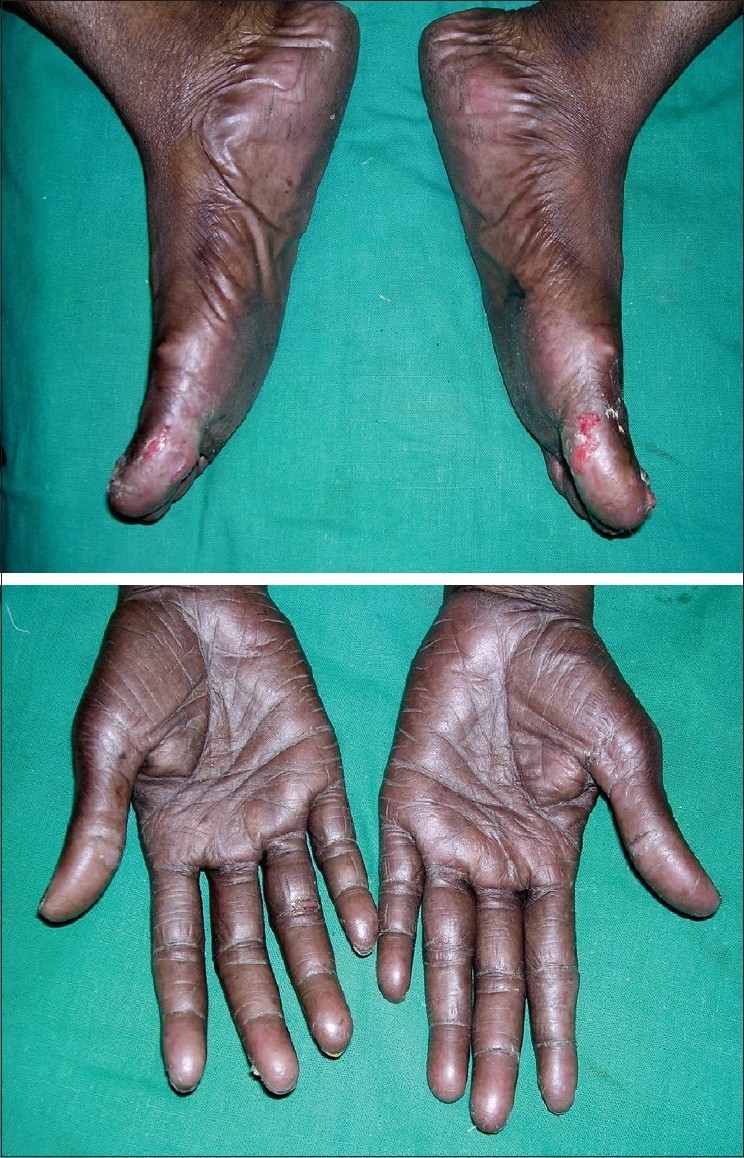
Dry, hyperpigmented palms and soles with ulceration over great toes with capecitabine therapy

In view of metastasis, patient was started on Tab. capecitabine (500 mg) 4 bid for 2 cycles. After the 2^nd^ cycle patient was referred to us for blackish discoloration of the palms and soles, painful shedding of nails, erosions and ulcerations on the medial aspect and ball of the great toes. On examination, patient had hyperpigmentation and dryness of skin with fissuring of palms and soles. There was loss of nails and ulcerations over the great toes bilaterally [Figure 1].

**Figure 2:**
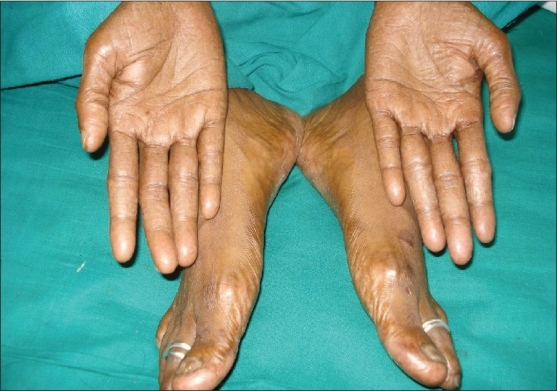
Resolution of dryness, pigmentation and ulceration after 3 months of stopping capecitabine

Patient was diagnosed as a case of hand and foot syndrome. Patient was treated with topical and systemic antibiotics. She was advised to put her hand in luke warm water daily for 5-10 min and liquid paraffin for local application. The dosage of Tab capecitabine (500mg) was reduced to 3 bid for 2 cycles followed by 3-0-2 for 4 cycles since the metastatic lesions were reduced. After stopping the drugs, the pigmentation and dryness decreases with resolution of ulceration in 3 months [Figure 2].

Capecitabine is a systemic prodrug of 5-fluorouracil (5-FU) and Hand-foot syndrome has proven to be a dose-limiting toxicity of capecitabine, leading to significant morbidity. The pathophysiology of Hand-foot syndrome is largely unknown. Histopathological changes include vacuolar degeneration of basal keratinocytes, dermal perivascular lymphocytic infiltration, apoptotic keratinocytes and dermal edema[3] Treatment include topical emollient, antibiotics to prevent secondary infection, topical steroid, Vitamin B_6_[4] and discontinuation of the offending drug in severe cases. In case of withdrawal, the offending drug may be cautiously re-introduced in a lower dose, which may gradually be stepped-up.
